# Relative Genetic and Environmental Contributions to Variations in Human Retinal Electrical Responses Quantified in a Twin Study

**DOI:** 10.1016/j.ophtha.2017.03.017

**Published:** 2017-08

**Authors:** Taha Bhatti, Ambreen Tariq, Ting Shen, Katie M. Williams, Christopher J. Hammond, Omar A. Mahroo

**Affiliations:** 1 Department of Ophthalmology, King's College London, St. Thomas' Hospital Campus, London, United Kingdom; 2 Department of Twin Research and Genetic Epidemiology, King's College London, St. Thomas' Hospital Campus, London, United Kingdom; 3 Retinal Service, Moorfields Eye Hospital, London, United Kingdom; 4 University College London Institute of Ophthalmology, London, United Kingdom; 5 Department of Physiology, Development and Neuroscience, University of Cambridge, Cambridge, United Kingdom

**Keywords:** ACE/ADE, components in twin modeling: additive genetic factors (A), common environment (C), dominant genetic factors (D), unique environment (E), ISCEV, International Society for the Clinical Electrophysiology of Vision, PhNR, photopic negative response

## Abstract

**Purpose:**

To estimate heritability of parameters of human retinal electrophysiology and to explore which parameters change with age.

**Design:**

Prospective, classic twin study.

**Participants:**

Adult monozygotic and dizygotic twin pairs recruited from the TwinsUK cohort.

**Methods:**

Electroretinogram responses were recorded using conductive fiber electrodes in response to stimuli incorporating standards set by the International Society for the Clinical Electrophysiology of Vision. These parameters were extracted; in addition, photopic negative-response (PhNR; originating from retinal ganglion cells) and i-wave components were extracted from responses to the photopic single flash. Parameter values were averaged from both eyes.

**Main Outcome Measures:**

Mean values were calculated for the cohort. Correlation coefficients with age were calculated (averaging parameters from both twins from each pair). Coefficients of intrapair correlation were calculated for monozygotic and dizygotic twins. Age-adjusted heritability estimates were derived using standard maximum likelihood structural equation twin modeling.

**Results:**

Responses were recorded from 210 participants in total (59 monozygotic and 46 dizygotic twin pairs). Ninety-three percent were women. Mean age for the cohort was 62.4 years (standard deviation, 11.4 years). In general, response amplitudes correlated negatively, and implicit times positively, with age. Correlations were statistically significant (*P* < 0.05) and moderate or strong (coefficient, >0.35) for the following parameters: scotopic standard and bright-flash a-wave implicit times, photopic 30-Hz flicker and single-flash b-wave implicit times, and PhNR and i-wave implicit times. Intrapair correlations were higher for monozygotic than dizygotic twins, suggesting important genetic influences. Age-adjusted estimates of heritability were significant for all parameters (except scotopic dim-flash b-wave implicit time), ranging from 0.34 to 0.85. Highest estimates were for photopic single-flash a-wave and b-wave amplitudes (0.84 and 0.85, respectively).

**Conclusions:**

This study explored heritability of retinal electrophysiologic parameters and included measurements reflecting ganglion cell function. Most parameters showed significant heritability, indicating that genetic factors are important, determining up to 85% of the variance in some cone system response parameters. Scotopic responses tended to show lower heritability (possibly relating to greater rod system susceptibility to environmental factors). Future studies can explore the identity of these genetic factors, improving our understanding of how they shape retinal function.

Impairments in function of retinal cell populations are an important cause of global visual impairment: Diabetes and inflammatory diseases frequently affect inner retinal neurons; age-related macular degeneration and inherited dystrophies affect outer retinal cells (photoreceptors, retinal pigment epithelium, and transmission to bipolar cells); and glaucoma, an important cause of sight loss worldwide, is a disease of retinal ganglion cells. In addition, myopia, whose prevalence is increasing worldwide, is now understood to be driven largely by retinal mechanisms.^1^ Our ability to image retinal anatomic features in vivo is advancing rapidly, with high-resolution imaging of retinal architecture widely available. This gives important information on structure, but does not always correlate completely with cellular function. Electroretinography, which is used less widely, allows objective, quantitative, noninvasive assessment of retinal function and can identify dysfunction before cell loss. In addition, because the synaptic pathways in the retina share features with excitatory and inhibitory pathways elsewhere in the brain, retinal electrophysiologic recordings can yield insights into pathogenetic mechanisms in neurologic conditions ranging from migraine^2^, ^3^ to schizophrenia^4^ and attention deficit–hyperactivity disorder.^5^

Studies in monozygotic and dizygotic twin pairs allow quantification of relative genetic and environmental contributions to variance in phenotypic traits. If the correlation of a phenotypic trait (or concordance of a disease) between monozygotic twins is higher than that between dizygotic twins, then the heritability (the proportion of the variance attributable to genetic factors) can be calculated (this is termed a *classic twin study*). Previous studies have shown that genetic factors make an important contribution to variance in retinal structure: macular thickness, as assessed by optical coherence tomography, has been estimated to have 81% to 85% heritability^6^; macular pigment density and patterns, as determined by 2-wavelength autofluorescence imaging, also are significantly heritable.^7^, ^8^, ^9^ However, there have been no large studies to date that directly explore heritability of electrophysiologic parameters of retinal function. A previous study in 42 twin pairs assessed aspects of visual function psychophysically, finding that processes involved in scotopic thresholds and adaptation may be more affected by environmental factors.^10^ Such psychophysical measurements relate to conscious perception, which is the culmination of layers of retinal and higher neuronal processing. This study aimed to quantify relative genetic and environmental contributions to visual function at the level of retinal cell signaling by measuring parameters of retinal electrophysiology in a significantly larger twin cohort. Correlations with age also were explored.

The International Society for the Clinical Electrophysiology of Vision (ISCEV) sets standards for full-field electroretinography,^11^ allowing assessment of generalized retinal function. These recordings permit distinction between diseases affecting rod and cone systems and also between disease processes affecting transduction in the photoreceptors and abnormalities of inner retinal processing. In the present study, more than 100 healthy twin pairs were recruited to undergo the full ISCEV electroretinography protocol. The parameters measured, as recommended by ISCEV, were a-wave and b-wave amplitudes and peak times for all flash stimuli, as well as amplitude and peak time of the photopic 30-Hz flicker. Figure 1 shows example traces, with these parameters labeled, as well as the likely cell populations from which the labeled components are thought to arise.

**Figure 1:**
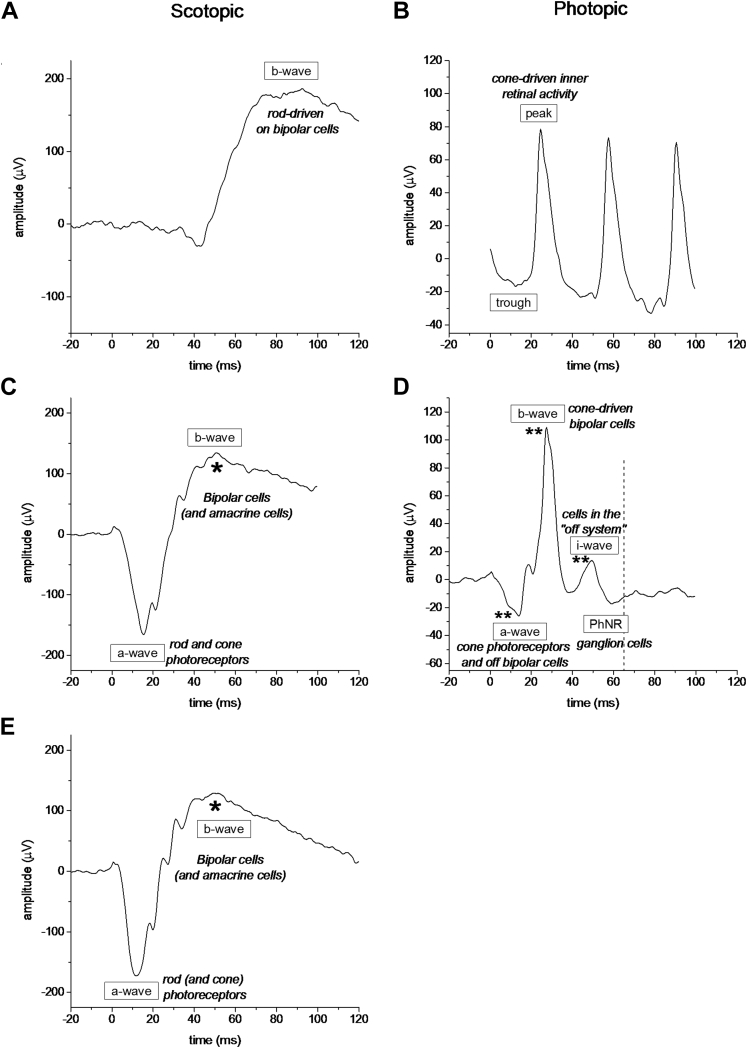
Graphs showing example electroretinography responses from a single participant to International Society for the Clinical Electrophysiology of Vision stimuli with labeling of parameters as well as their cellular origin. **A**, **C**, **E**, Averaged responses to white flashes delivered in the dark after 20 minutes of dark adaptation: (**A**) 0.01 cd s/m^2^. (**C**) 3.0 cd s/m^2^, and (**E**) 10.0 cd s/m^2^. **B**, **D**, Averaged responses to stimuli delivered after 10 minutes of adaptation to a 30-cd s/m^2^ white background: (**B**) Response to 30-Hz flicker and (**D**) response to 3.0-cd s/m^2^ flash. Additional parameters (photopic negative response [PhNR] and i-wave) are labeled in (**D**). *Asterisks* denote components whose amplitudes were found to have high heritability as estimated in this study. ^∗^Denotes point estimate of heritability of more than 0.75, which was the case for scotopic b-wave amplitudes in response to the brighter flashes. ^∗∗^Denotes heritability point estimates of more than 0.80, which was the case for photopic flash a-wave, b-wave, and i-wave amplitudes.

A later negative component of photopic (light-adapted) flash electroretinography, occurring 65 to 75 ms after flash delivery, was identified 17 years ago as likely to be arising from retinal ganglion cells; it was abolished by experimentally induced glaucoma in macaques.^12^ It was termed the *photopic negative response* (PhNR), and more than 100 publications since have explored features of this component, in particular its possible usefulness in evaluating dysfunction of the retinal ganglion cells in assessment of glaucoma. In view of the potential future clinical importance of this parameter, we identified the component in recordings from our twin participants (Fig 1) and explored its heritability in addition to the ISCEV parameters listed above. An additional electroretinography component just preceding the PhNR, the i-wave, which may originate from the OFF pathway distal to retinal ganglion cells,^13^ also was identified and investigated.

## Methods

### Participants

Participants were recruited from the TwinsUK cohort, based at St. Thomas' Hospital, London. This cohort comprises approximately 12 000 adult twins (83% women) from the United Kingdom who have volunteered to participate in research studies.^14^ Both members of each twin pair attended together, and recordings were performed consecutively, first on one twin and then the fellow twin. In the case of 1 pair, the 2 twins attended on separate days, but recordings were performed at the same time of day. Participants were asked about any eye conditions before recording. Pupils were dilated pharmacologically with mydriatic drops (1.0% tropicamide and, in most cases, 2.5% phenylephrine). Both members of each twin pair were given the same dilating drops (i.e., if one twin received only tropicamide, then so did the other twin).

### Stimuli

Stimuli were delivered, and responses recorded, using the Diagnosys Colordome with Espion software (Diagnosys, Lowell, MA). Stimuli corresponded to the ISCEV standard for full-field electroretinography.^11^ Participants underwent a minimum of 20 minutes of dark adaptation before the delivery of scotopic flash stimuli (white flashes, delivering 0.01, 3.0, and 10.0 cd s/m^2^ photopic light). Participants underwent a minimum of 10 minutes of light adaptation (to the ISCEV white adapting background of 30 cd s/m^2^ photopic light) before the delivery of photopic stimuli, which included the 30-Hz flicker and the photopic single flash (both 3.0 cd s/m^2^ photopic light). Stimuli were presented repeatedly and responses were averaged. After the scotopic stimuli described previously, and before the ISCEV light adaptation period, additional flash stimuli were presented both in the dark and on a rod-saturating blue background to explore additional parameters of photoreceptor function (analysis not described here). Also, during the light adaptation period, additional photopic flicker and flash stimuli (corresponding to ISCEV standard photopic stimuli) were delivered. The responses to the flash stimuli delivered throughout this period were used to extract the PhNR component.

### Recording

Electroretinography recordings were made from both eyes using a conductive fiber electrode (DTL-PLUS electrode; Unimed Electrode Supplies Limited, Farnham, Surrey, United Kingdom) placed consistently in the lower conjunctival fornix. Because electrode position can affect amplitude of electroretinography responses (Tariq A, et al., *Invest Ophthalmol Vis Sci.* 55[13], 2014; ARVO E-Abstract 5121), the location was checked regularly during recordings, and if necessary, the electrode was repositioned into the fornix. The indifferent electrode was placed at the temple and a ground electrode was placed on the forehead. These were skin-surface electrodes (24-mm disposable ground electrodes; Unimed Electrode Supplies Limited), placed after cleaning of the skin with alcohol wipes.

### Response Parameters

Stimuli were presented multiple times and responses were averaged after rejection of traces contaminated by excessive noise or blink artefact (criteria for trace rejection were similar to those described previously^15^). The following 16 ISCEV parameters were recorded for each eye (Fig 1): a-wave amplitudes and implicit times for scotopic standard and bright flashes and for the photopic single flash; b-wave amplitudes and implicit times for scotopic dim, standard, and bright flashes and for the photopic single flash; and peak amplitude and implicit time for the photopic 30-Hz flicker response. Also, the b-wave–to–a-wave amplitude ratio was calculated for flash stimuli. Because the photopic responses are lower in amplitude, with a relatively poorer signal-to-noise ratio, greater numbers of responses were averaged (up to 60).

For extraction of the PhNR, the photopic single-flash response was used. To improve the signal-to-noise ratio, traces were averaged from additional flashes delivered during the light adaptation period (these were after prior adaptation to the rod-saturating blue background, and hence the retina was already relatively light adapted). A total of 180 responses were averaged. The PhNR was measured in 2 different ways: first, the amplitude of the response measured at a fixed time (65 milliseconds) after flash delivery was recorded as in previous investigations,^16^ and second, the amplitude and timing of the trough after the i-wave was recorded (Fig 1). These amplitudes were measured relative to the preflash baseline. The ratio of the amplitude of this trough to the b-wave amplitude also was calculated because this ratio has been suggested to be less variable and potentially clinically more useful.^17^ In addition, the amplitude and timing of the i-wave, where identifiable, was recorded.

### Heritability Analysis

For investigation of heritability, response parameters for right and left eye were averaged for each participant and adjusted linearly for the effect of age (because mean ages differed for monozygotic and dizygotic pairs). Coefficients for intrapair correlation were calculated for monozygotic and dizygotic twins. Heritability was calculated formally for each of the parameters described above. These calculations were performed with maximum likelihood structural equation twin modeling as described previously,^18^ using the OpenMx package (http://openmx.psyc.virginia.edu) in the R statistical computing environment (http://www.r-project.org). In this method, the variance of a trait is estimated by the contributions of some combination of 3 factors—the additive genetic component (A), the shared environment (C), or the nonadditive genetic component (D)—and the unique environment (E). Univariant ACE or ADE models were executed with standardized path coefficients and expected variance and covariance matrices. The goodness of fit of the full and reduced ACE and ADE models were compared with the observed data. The most parsimonious model to explain the observed variance was selected using the Akaike information criterion; this was identified as the AE model for most traits. Heritability was calculated as the proportion of total variance of the trait (V) resulting from the additive genetic effect (A) in the best-fitting model.

### Correlations with Age

Coefficients of correlation with age were calculated for each parameter. Because measurements from members of a twin pair are likely to be correlated, averaged parameters per twin pair were included for this calculation (so each pair was included only once).

### Ethical Approval

Participants gave informed consent. The study had local research ethics committee approval and was conducted in accordance with the tenets of the Declaration of Helsinki.

### Literature Search for Previous Studies

We performed a literature search in the PubMed database (https://www.ncbi.nlm.nih.gov/pubmed/; accessed February 28, 2017) to identify any previous electroretinography twin studies (using search terms including *electroretinogram* and *twins*).

## Results

Responses were recorded from 210 participants in total (59 monozygotic and 46 dizygotic complete twin pairs). One hundred ninety-six participants (93%) were women. Two hundred four participants (97%) were of white European ancestry. Most participants (>90%) did not report any eye condition expected to affect the electroretinography results. Eighteen participants were noted to have the following: age-related macular degeneration (4 individuals), diabetes (5 individuals), previous retinal detachment (2 individuals), unspecified retinal problems (2 individuals), glaucoma (3 individuals), and glaucoma suspect (2 individuals). Mean age for the cohort was 62.4 years (standard deviation, 11.4 years); the median age was 64.3 years. The age distribution is shown in Figure S1 (available at www.aaojournal.org). The mean ages for monozygotic and dizygotic twins were 60.5 years (standard deviation, 11.2 years) and 65.0 years (standard deviation, 11.1 years), respectively, with monozygotic pairs being significantly younger on average by 4.5 years (*P* = 0.0044).

### Mean International Society for the Clinical Electrophysiology of Vision Parameter Values and Correlation with Age

**Figure 2:**
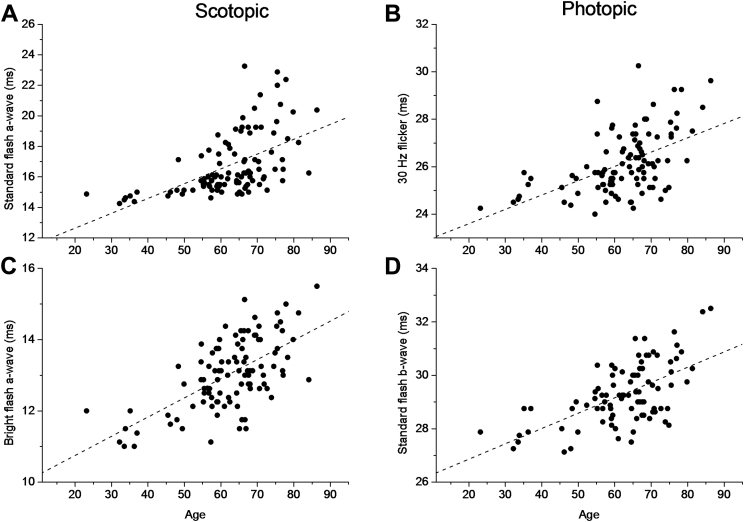
Plots of International Society for the Clinical Electrophysiology of Vision response parameters found to correlate significantly with age. *Dashed lines* show a simple fit by linear regression. **A**, Scotopic standard flash a-wave implicit time. **B**, Scotopic bright flash a-wave implicit time. **C**, Photopic 30 Hz flicker response peak time. **D**, Photopic standard flash b-wave implicit time.

Table 1 shows mean ISCEV parameter values for the entire cohort and their correlation with age. In all cases, amplitudes showed negative correlations with age, whereas implicit times showed positive correlations, consistent with older participants having smaller and more delayed responses. However, most correlations were weak. Correlations were statistically significant for the following parameters: scotopic dim-flash b-wave amplitude and implicit time; scotopic standard- and bright-flash a-wave and b-wave implicit times; photopic 30-Hz flicker amplitude and implicit time; and photopic single-flash a-wave and b-wave amplitudes (including b-to-a ratio) and implicit times. Of these, correlations were moderately strong (coefficient, >0.5) for the following: scotopic standard- and bright-flash a-wave implicit times and photopic 30-Hz flicker and single-flash b-wave implicit times. Figure 2 plots these parameters as a function of age; from a simple linear fit, the average increase in implicit time per decade was found to be 0.97, 0.54, 0.60, and 0.57 ms for the 4 parameters, respectively.

**Table 1:** Mean Parameter Values and Standard Deviations for International Society for the Clinical Electrophysiology of Vision Stimuli for the Entire Cohort and Correlations with Age

Stimulus	Response Component	Parameter	Parameter Values		Correlation with Age (Spearman)	
			Mean (Standard Deviation)	No.	Coefficient	*P* Value
Scotopic dim flash	b-wave	Amplitude	184.6 (43.6)	206	−0.26	0.007∗
		Implicit time	97.9 (11.7)	206	0.28	0.004∗
Scotopic standard flash	a-wave	Amplitude	−145.4 (32.6)	206	−0.19	0.059
		Implicit time	16.8 (2.4)	206	0.62	2.7 × 10^−12^∗
	b-wave	Amplitude	262.4 (57.1)	206	−0.19	0.059
		Implicit time	52.1 (3.3)	206	0.36	1.6 × 10^−4^∗
	b-to-a ratio		1.83 (0.30)	206	0.12	0.228
Scotopic bright flash	a-wave	Amplitude	−172.3 (35.6)	205	−0.16	0.104
		Implicit time	13.0 (1.1)	205	0.56	7.1 × 10^−10^∗
	b-wave	Amplitude	272.5 (56.5)	205	−0.16	0.103
		Implicit time	50.2 (4.0)	205	0.23	0.021∗
	b-to-a ratio		1.60 (0.23)	205	0.07	0.500
Photopic 30-Hz flicker	Peak	Amplitude	69.7 (19.3)	194	−0.21	0.031∗
		Implicit time	26.2 (1.4)	194	0.51	3.4 × 10^−8^∗
Photopic single flash	a-wave	Amplitude	−22.1 (5.5)	178	−0.23	0.032∗
		Implicit time	14.3 (0.64)	178	0.31	0.003∗
	b-wave	Amplitude	95.0 (25.2)	178	−0.33	0.001∗
		Implicit time	29.3 (1.3)	178	0.53	1.0 × 10^−7^∗
	b-to-a ratio		4.34 (0.70)	178	−0.22	0.033∗

The amplitudes are in microvolts and times in milliseconds. Some responses were excluded, for example because of noise artefacts, so the total numbers of participants differed for some parameters and are given in the number column. For correlations with age, the Spearman correlation coefficient was used, because tests for normality (Shapiro-Wilks) showed that most parameters were not normally distributed. Also, for age correlations, the mean parameter value from both twins in each pair was included to avoid confounding because of intrapair correlation. Hence, the numbers included are approximately half of the entire cohort. Correlations for amplitudes relate to magnitude (regardless of positive or negative sign), and hence a negative correlation for the a-wave or b-wave indicates a smaller amplitude in older participants.

∗ *P* < 0.05 was regarded as statistically significant.

### Intrapair Correlations and Heritability Estimates for International Society for the Clinical Electrophysiology of Vision Parameters

**Figure 3:**
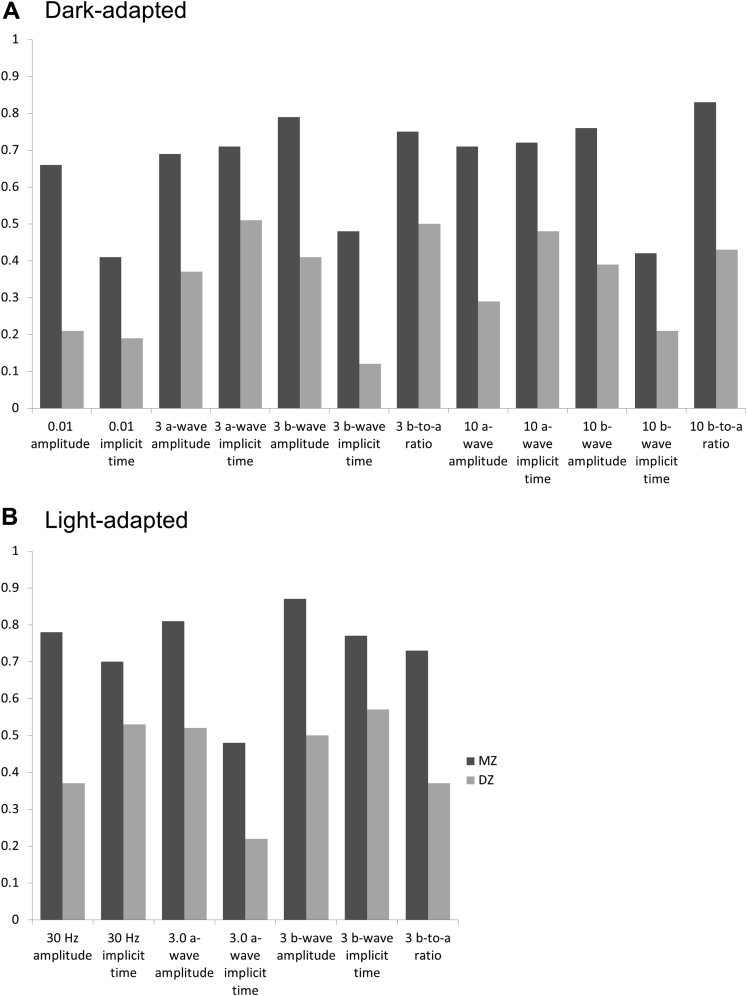
Bar graphs showing coefficients of intrapair correlation for monozygotic and dizygotic twins for International Society for the Clinical Electrophysiology of Vision parameters. (**A**, scotopic stimuli; **B**, photopic stimuli). In labels to the bars, numbers without units refer to the standard flash intensities in photopic cd s/m^2^. DZ = dizygotic; MZ = monozygotic.

Table 2 shows coefficients of intrapair correlation for monozygotic and dizygotic twins for each parameter. Correlations were significant for all parameters in monozygotic twins and for most parameters in dizygotic twins. Figure 3 depicts the correlations; in all cases, correlations were higher for monozygotic twins. Age-adjusted heritability was calculated, and estimates with 95% confidence intervals are shown in the final column of Table 2. In most cases, the AE model provided the most parsimonious fit. For 2 parameters (listed in the legend), the fit was marginally better with the ADE model. Full details relating to goodness of fit can be found in Table S1 (available at www.aaojournal.org). Heritability was highest for photopic single-flash a-wave and b-wave amplitudes.

**Table 2:** Coefficients for Intrapair Correlation for Monozygotic and Dizygotic Twins and Estimates of Heritability for International Society for the Clinical Electrophysiology of Vision Parameters

Stimulus	Response Component	Parameter	Monozygotic Pairs		Dizygotic Pairs		Heritability (95% Confidence Interval)
			Coefficient	*P* Value	Coefficient	*P* Value	
Scotopic dim flash	b-wave	Amplitude	0.66	2.2 × 10^−8^∗	0.21	0.164	0.64 (0.48–0.76)
		Implicit time	0.41	0.0014∗	0.19	0.217	0.12 (<0.01–0.34)†
Scotopic standard flash	a-wave	Amplitude	0.69	3.1 × 10^−9^∗	0.37	0.011∗	0.69 (0.55–0.79)
		Implicit time	0.71	43 × 10^−10^∗	0.51	2.6 × 10^−4^∗	0.61 (0.39–0.75)‡
	b-wave	Amplitude	0.79	1.7 × 10^−13^∗	0.41	0.005∗	0.79 (0.69–0.86)
		Implicit time	0.48	1.4 × 10^−4^∗	0.12	0.435	0.48 (0.27–0.65)
	b-to-a ratio		0.75	1.43 × 10^−11^∗	0.50	4.4 × 10^−4^∗	0.69 (0.54–0.79)
Scotopic bright flash	a-wave	Amplitude	0.71	8.8 × 10^−10^∗	0.29	0.054	0.71 (0.57–0.81)
		Implicit time	0.72	3.5 × 10^−10^∗	0.48	8.1 × 10^−4^∗	0.57 (0.36–0.72)
	b-wave	Amplitude	0.76	1.8 × 10^−11^∗	0.39	0.008∗	0.77 (0.66–0.85)
		Implicit time	0.42	0.0011∗	0.21	0154	0.51 (0.30–0.67)‡
	b-to-a ratio		0.83	4.2 × 10^−15^∗	0.43	0.003∗	0.81 (0.71–0.88)
Photopic 30-Hz flicker	Peak	Amplitude	0.78	9.2 × 10^−12^∗	0.37	0.019∗	0.64 (0.49–0.76)
		Implicit time	0.70	9.0 × 10^−9^∗	0.53	4.5 × 10^−4^∗	0.61 (0.40–0.75)
Photopic single flash	a-wave	Amplitude	0.81	1.7 × 10^−12^∗	0.52	6.5 × 10^−4^∗	0.84 (0.75–0.90)
		Implicit time	0.48	46 × 10^−4^∗	0.22	0.174	0.34 (0.08–0.55)
	b-wave	Amplitude	0.87	4.4 × 10^−16^∗	0.50	0.001∗	0.85 (0.76–0.90)
		Implicit time	0.77	9.9 × 10^−11^∗	0.57	1.6 × 10^−4^∗	0.65 (0.47–0.78)
	b-to-a ratio		0.73	2.0 × 10^−9^∗	0.37	0.019∗	0.79 (0.66–0.86)

For all but 3 parameters (listed below), the AE (additive genetic factors; unique environment) model provided the best fit.

∗ *P* < 0.05 was considered statistically significant.

† For this parameter (scotopic dim-flash b-wave implicit time), the E model gave a marginally better fit, suggesting that the variance in this timing could be attributable entirely to unique environmental factors including measurement error.

‡ For these 2 parameters, the ADE model gave a marginally better fit, with heritability largely accounted for by (D) additive and dominant genetic factors. The estimates of heritability were 0.61 (95% confidence interval, 0.39–0.75) and 0.54 (95% confidence interval, 0.34–0.69) for the implicit times of the scotopic standard flash a-wave and scotopic bright flash b-wave, respectively.

### Mean Photopic Negative Response and i-Wave Parameter Values and Correlation with Age

**Figure 4:**
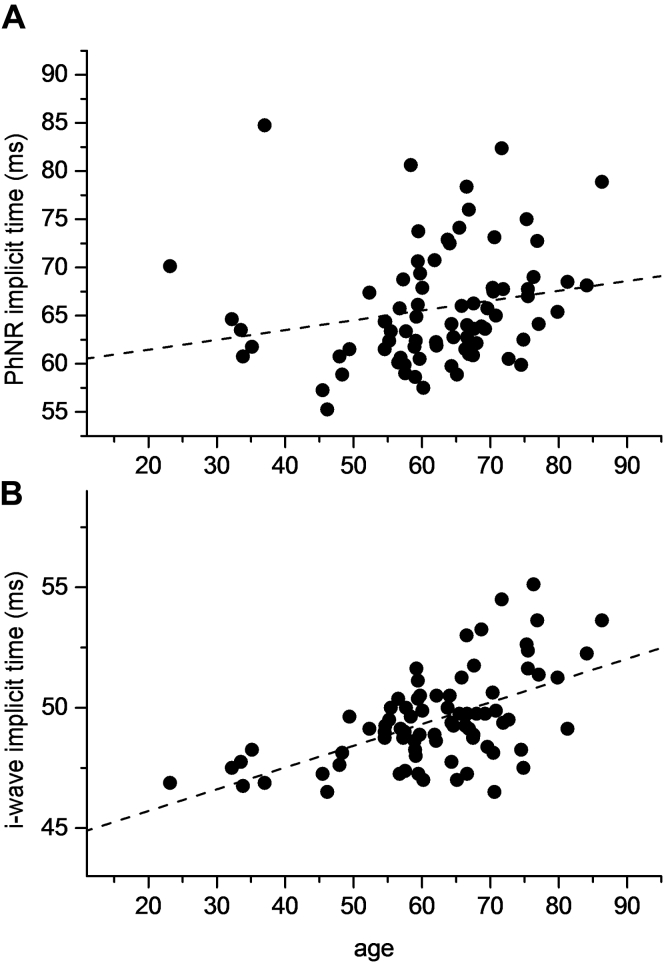
Scatterplots showing (**A**) photopic negative-response (PhNR) implicit time and (**B**) i-wave implicit time against age (as derived from the response to the International Society for the Clinical Electrophysiology of Vision standard photopic flash). *Dashed lines* show a simple fit by linear regression.

Table 3 shows mean parameter values for the photopic negative response (measured in different ways as detailed in “Methods”) and i-wave. There appeared to be little change in amplitudes with age, but implicit times for both parameters correlated significantly with age, with older participants showing increased delay (Fig 4). By linear regression, the increase in implicit time per decade was 1.02 and 0.90 milliseconds for PhNR and i-wave, respectively. Because the PhNR is expected to be affected by glaucoma, means were recalculated after exclusion of participants who reported having a diagnosis of glaucoma or possible glaucoma (glaucoma suspects). Because there were very few such participants (only 2% of the cohort), the mean values were found to be unchanged (to the degree of accuracy of the figures in Table 3).

**Table 3:** Mean Parameter Values and Standard Deviations for Photopic Negative Response and i-Wave for the Entire Cohort and Correlations with Age

Response Component	Parameter	Parameter Values		Correlation with Age (Spearman)	
		Mean (Standard Deviation)	No.	Coefficient	*P* Value
PhNR	Amplitude at 65 ms	−15.2 (4.2)	158	−0.19	0.097
	Amplitude at trough	−17.6 (4.8)	158	−0.28	0.013∗
	Implicit time of trough	65.7 (7.0)	158	0.33	0.003∗
	PhNR–to–b-wave ratio	0.20 (0.06)	134	0.05	0.691
i-wave	Amplitude	1.13 (5.54)	158	0.10	0.361
	Implicit time	49.5 (2.1)	158	0.51	1.5 × 10^−6^∗

PhNR = photopic negative response.

Amplitudes are in microvolts (compared with baseline before flash) and times are in milliseconds. Correlations for amplitudes relate to magnitude (irrespective of positive or negative sign) and hence a negative correlation indicates a smaller amplitude in older subjects.

∗ *P* < 0.05 was considered statistically significant.

### Intrapair Correlations and Heritability Estimates for Photopic Negative Response and i-Wave

**Figure 5:**
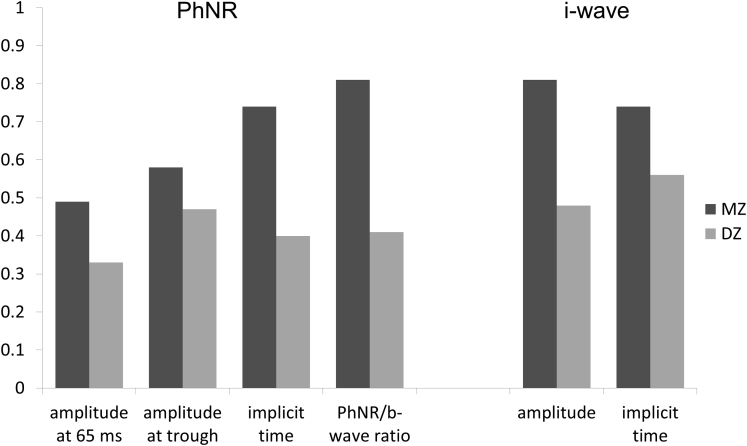
Bar graphs showing coefficients of intrapair correlation for parameters relating to the photopic negative response (PhNR) and i-wave for monozygotic (MZ) and dizygotic (DZ) twins (as derived from the response to the International Society for the Clinical Electrophysiology of Vision standard photopic flash).

Table 4 shows coefficients of intrapair correlation for monozygotic and dizygotic twins (demonstrated graphically in Fig 5). Age-adjusted heritability estimates are shown in the final column. In all cases, the AE model provided the most parsimonious fit. Data regarding goodness of fit are included in Table S1 (available at www.aaojournal.org). All parameters showed significant heritability, with the lower limit of the confidence interval higher than 50% for PhNR implicit time and PhNR–to–b-wave ratio as well as for i-wave amplitude.

**Table 4:** Coefficients for Intrapair Correlation for Monozygotic and Dizygotic Twins and Estimates of Heritability for Photopic Negative Response and i-Wave

Response Component	Parameter	Monozygotic Pairs		Dizygotic Pairs		Heritability (95% Confidence Interval)
		Coefficient	*P* Value	Coefficient	*P* Value	
PhNR	Amplitude at 65 ms	0.49	9.19 × 10^−4^∗	0.33	0.0511	0.55 (0.31–0.72)
	Amplitude at trough	0.58	3.92 × 10^−5^∗	0.47	0.0037∗	0.65 (0.45–0.78)
	Implicit time of trough	0.74	1.87 × 10^−8^∗	0.40	0.0146∗	0.74 (0.56–0.84)
	PhNR/b-wave	0.81	1.74 × 10^−9^∗	0.41	0.0233∗	0.69 (0.52–0.80)
i-wave	Amplitude	0.81	3.84 × 10^−11^∗	0.48	0.0030∗	0.83 (0.72–0.89)
	Implicit time	0.74	1.86 × 10^−8^∗	0.56	3.65 × 10^−4^∗	0.62 (0.40–0.76)

PhNR = photopic negative response.

∗ *P* < 0.05 considered statistically significant.

### Results of Literature Search

Our literature search identified a single previous twin study of electroretinography parameters.^19^ This study, published in 1960 (before the establishment of international standards for stimulus parameters), reported findings from measuring b-wave amplitudes in 14 twin pairs and found that variability between twin pairs was greater than that within pairs and that this was more so for nonidentical than identical twins, although no formal heritability estimation was attempted.

## Discussion

This study aimed to quantify genetic contributions to the variance in parameters of retinal electrophysiologic function by recording electroretinography responses to international standard scotopic and photopic full-field stimuli in a cohort of more than 100 twin pairs. All response parameters were found to have higher intrapair correlations in monozygotic compared with dizygotic twins, indicating an importance of genetic factors. When heritability was estimated explicitly by twin modeling, all parameters (other than the scotopic dim-flash b-wave implicit time) showed significant heritability, with point estimates ranging from 34% to 84%. In addition, parameters relating to the photopic negative response, originating from retinal ganglion cells and of interest in relation to ganglion cell diseases including glaucoma, showed high heritability, as did parameters relating to the i-wave, a small peak in the photopic response waveform shortly after the b-wave.

With regard to ISCEV parameters, the highest heritability was found for the photopic single-flash a-wave and b-wave amplitudes, with point estimates of 0.84 and 0.85, respectively. The photopic single-flash a-wave originates from cone photoreceptors and cone-OFF bipolar cells^20^; the photopic b-wave is shaped by synchronous changes in current flows in ON and OFF bipolar cells. The finding of greater heritability in photopic responses may suggest that scotopic function is relatively more susceptible to environmental influences, which may relate to greater vulnerability of rod function in a number of disease processes (e.g., the impairment of scotopic dark adaptation that occurs in age-related maculopathy). The photopic single-flash b-wave amplitude is altered in conditions affecting the cone system, and intriguingly was found to be reduced significantly in a recent study of individuals with autism spectrum disorder.^21^ Autism spectrum disorder itself has been shown to be significantly heritable.^22^

Estimates of heritability tended to be higher for response amplitudes than for implicit times, with almost all amplitudes having a lower limit of the 95% confidence interval for heritability that exceeded 50%. This is intriguing because implicit times tend to show less intersession and interlaboratory variability than response amplitudes,^23^ suggesting that measurement error would be greater in amplitude quantification rather than implicit time. Measurement errors act as individual (i.e., unique) environmental factors in twin studies, and so would reduce estimates of heritability. Thus, the high heritability of response amplitudes compared with implicit times lends weight to the notion that the former may have greater genetic influence. Implicit time heritability was relatively high for photopic flicker and flash b-wave responses, consistent with the notion that both parameters may derive from a common source (cone-driven signals that originate in the inner rather than outer retina), whereas the photopic flash a-wave implicit time heritability was lower. The lowest implicit time heritability was for the scotopic dim-flash b-wave implicit time. This signal, originating in rod-driven ON bipolar cells, is a somewhat slow waveform, and the specific time of the peak can be highly dependent on superimposed high-frequency noise. It is possible that low-pass filtering or the fitting of a smooth curve to the response may reduce this variability.

This study also explored changes in these parameters with age. This is informative because it provides insight into how retinal signaling processes may change with age and also in defining what is considered to be normal in different age groups, enhancing the usefulness of electroretinography in detecting clinically significant abnormalities. Amplitudes were correlated negatively with age and implicit times were correlated positively with age, consistent with a reduction in magnitude as well as a slowing of retinal electrical responses with age. In many cases, the strength of the correlation seemed to be greater for implicit times than for amplitudes, suggesting that slowing with age may be more significant. During the course of one's lifetime, environmental factors may gain in relative importance compared with genetic factors, although the two are clearly interlinked; this may be consistent with implicit times showing lower heritability, but stronger correlation with age. Previous studies also have identified different normative ranges for different age groups for ISCEV parameters,^24^ and the findings of the present study are broadly consistent with the previous trends identified. This study gives estimates of change per decade in the more strongly age-correlated parameters. However, it should be noted that this is somewhat simplistic, assuming a monotonic linear variation with age, where the true relationship may be more complex. Cursory visual inspection of the data plotted in Figures 2 and 4 also may suggest that a relatively flat or shallow relationship with age exists in younger age groups, and this becomes more steep in older decades, together with an increasing variability in older age groups.

The present study also investigated additional parameters of the photopic single-flash response (not currently defined by ISCEV) that may be of increasing interest. The b-wave is followed by a small peak, termed the *i-wave*, and a subsequent trough (whose full waveform is probably interrupted by the i-wave peak), termed the *PhNR*. The i-wave is thought to originate in the so-called OFF system somewhere distal to the retinal ganglion cells, whereas the PhNR seems to come from the retinal ganglion cells themselves.^13^ Different stimuli for eliciting the PhNR have been described, in terms of characteristics of both stimulus (duration, intensity, and spectral composition) and background (intensity and spectral composition).^16^ This study used the standard ISCEV photopic single flash (a 3.0-cd s/m^2^ photopic white flash delivered on a 30-cd s/m^2^ photopic white background through a pharmacologically dilated pupil). It has been suggested that optimal stimuli for PhNR quantification are those stimulating 1 cone type on a background with minimal cone adaptive effects, such as a red flash on a blue background.^16^ Our study provides a normative range in a large cohort for the PhNR and i-wave as derived from the standard photopic white flash. The mean and standard deviations are given in Table 3. Implicit times showed significant positive correlation with age. Intrapair coefficients of correlation were greater in monozygotic than dizygotic twins, and significant heritability was demonstrated. Point estimates of heritability of PhNR amplitudes were slightly higher when this was normalized to the b-wave. Previous studies have suggested that normalizing to the b-wave may reduce the variability in this parameter,^25^ and the finding of greater heritability would support this (because this could result from reduced measurement error). Interestingly, the i-wave amplitude, although its precise origin is not clear, had a high estimated heritability of 83% (Fig 5).

Some limitations of the study deserve mention. The relative homogeneity of the cohort demographics (mostly women and those of Northern European descent) may make the findings less generalizable to wider populations and other age ranges. Some age ranges were overrepresented, and so the age-related correlations should be interpreted in light of this; it is possible that a study recruiting, from the outset, equal numbers of participants across different age groups would reveal stronger or more significant correlations. In addition, although participants were asked about eye problems, all participants did not undergo a full dilated eye examination, and so some retinal pathologic features that may affect electroretinography responses might have been undetected.

In summary, our study has shown that multiple parameters of retinal electrophysiologic function, as recorded by electroretinography in both scotopic and photopic conditions, are significantly heritable, with up to 85% of the variance being attributable to genetic factors in some cases. Future studies may explore the identity of the genetic factors shaping retinal function, which would enhance our understanding of retinal signaling in health and its alteration in disease.

## References

[1] Flitcroft D.I. (2012). The complex interactions of retinal, optical and environmental factors in myopia aetiology. Prog Retin Eye Res.

[2] Noseda R., Bernstein C.A., Nir R.R. (2016). Migraine photophobia originating in cone-driven retinal pathways. Brain.

[3] Verroiopoulos G.V., Nitoda E., Ladas I.D. (2016). Ophthalmological assessment of OCT and electrophysiological changes in migraine patients. J Clin Neurophysiol.

[4] Hébert M., Mérette C., Paccalet T. (2015). Light evoked potentials measured by electroretinogram may tap into the neurodevelopmental roots of schizophrenia. Schizophr Res.

[5] Bubl E., Dörr M., Riedel A. (2015). Elevated background noise in adult attention deficit hyperactivity disorder is associated with inattention. PLoS One.

[6] Chamberlain M.D., Guymer R.H., Dirani M. (2006). Heritability of macular thickness determined by optical coherence tomography. Invest Ophthalmol Vis Sci.

[7] Liew S.H., Gilbert C.E., Spector T.D. (2005). Heritability of macular pigment: a twin study. Invest Ophthalmol Vis Sci.

[8] Hogg R.E., Ong E.L., Chamberlain M. (2012). Heritability of the spatial distribution and peak density of macular pigment: a classical twin study. Eye (Lond).

[9] Tariq A., Mahroo O.A., Williams K.M. (2014). The heritability of the ring-like distribution of macular pigment assessed in a twin study. Invest Ophthalmol Vis Sci.

[10] Hogg R.E., Dimitrov P.N., Dirani M. (2009). Gene-environment interactions and aging visual function: a classical twin study. Ophthalmology.

[11] McCulloch D.L., Marmor M.F., Brigell M.G. (2015). ISCEV Standard for full-field clinical electroretinography (2015 update). Doc Ophthalmol.

[12] Viswanathan S., Frishman L.J., Robson J.G. (1999). The photopic negative response of the macaque electroretinogram: reduction by experimental glaucoma. Invest Ophthalmol Vis Sci.

[13] Rangaswamy N.V., Frishman L.J., Dorotheo E.U. (2004). Photopic ERGs in patients with optic neuropathies: comparison with primate ERGs after pharmacologic blockade of inner retina. Invest Ophthalmol Vis Sci.

[14] Moayyeri A., Hammond C.J., Hart D.J., Spector T.D. (2013). The UK Adult Twin Registry (TwinsUK Resource). Twin Res Hum Genet.

[15] Paupoo A.A., Mahroo O.A., Friedburg C., Lamb T.D. (2000). Human cone photoreceptor responses measured by the electroretinogram [correction of electoretinogram] a-wave during and after exposure to intense illumination. J Physiol.

[16] Rangaswamy N.V., Shirato S., Kaneko M. (2007). Effects of spectral characteristics of Ganzfeld stimuli on the photopic negative response (PhNR) of the ERG. Invest Ophthalmol Vis Sci.

[17] Fortune B., Bui B.V., Cull G. (2004). Inter-ocular and inter-session reliability of the electroretinogram photopic negative response (PhNR) in non-human primates. Exp Eye Res.

[18] Mahroo O.A., Oomerjee M., Williams K.M. (2014). High heritability of posterior corneal tomography, as measured by Scheimpflug imaging, in a twin study. Invest Ophthalmol Vis Sci.

[19] Ziv B. (1960). The electroretinogram (ERG) in human twins. A comparative statistical evaluation of the “b-wave” of identical and fraternal twins. Arch Ophthalmol.

[20] Robson J.G., Saszik S.M., Ahmed J., Frishman L.J. (2003). Rod and cone contributions to the a-wave of the electroretinogram of the macaque. J Physiol.

[21] Constable P.A., Gaigg S.B., Bowler D.M. (2016). Full-field electroretinogram in autism spectrum disorder. Doc Ophthalmol.

[22] Tick B., Bolton P., Happé F. (2016). Heritability of autism spectrum disorders: a meta-analysis of twin studies. J Child Psychol Psychiatry.

[23] Hamilton R., Al Abdlseaed A., Healey J. (2015). Multi-centre variability of ISCEV standard ERGs in two normal adults. Doc Ophthalmol.

[24] Neveu M.M., Dangour A., Allen E. (2011). Electroretinogram measures in a septuagenarian population. Doc Ophthalmol.

[25] Mortlock K.E., Binns A.M., Aldebasi Y.H., North R.V. (2010). Inter-subject, inter-ocular and inter-session repeatability of the photopic negative response of the electroretinogram recorded using DTL and skin electrodes. Doc Ophthalmol.

