# Supplementary material for: Relative Genetic and Environmental Contributions to Variations in Human Retinal Electrical Responses Quantified in a Twin Study
Source: Ophthalmology. 2017 Aug;124(8):1175–85. doi: 10.1016/j.ophtha.2017.03.017 (PMC5540060; doi:10.1016/j.ophtha.2017.03.017)

Supplementary Figure. Age distribution of the cohort. Numbers of individuals in each decade are shown above the bars.

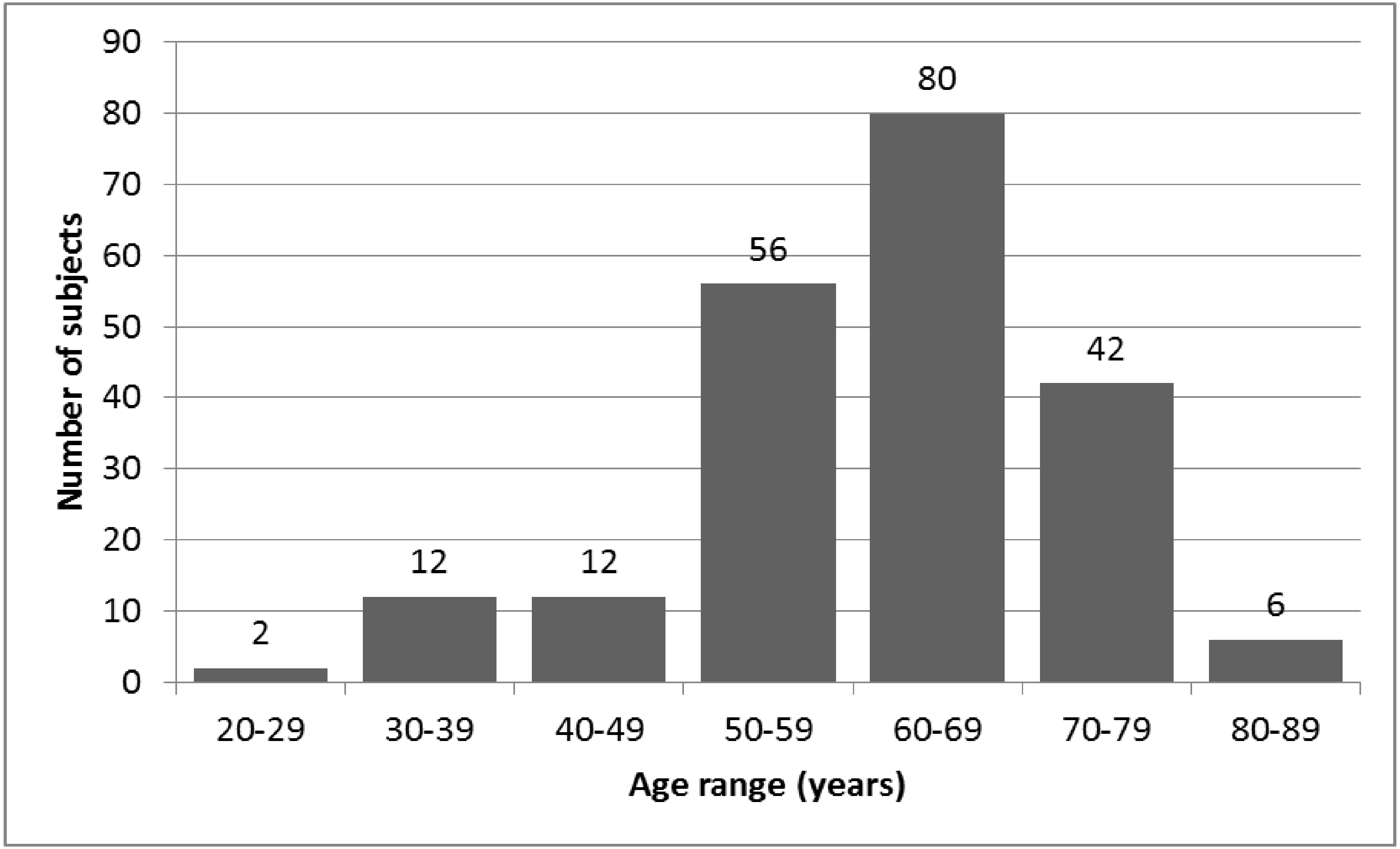

Supplement: Figure S1 [file mmc1.pdf]
