# Supplementary material for: Relative Genetic and Environmental Contributions to Variations in Human Retinal Electrical Responses Quantified in a Twin Study
Source: Ophthalmology. 2017 Aug;124(8):1175–85. doi: 10.1016/j.ophtha.2017.03.017 (PMC5540060; doi:10.1016/j.ophtha.2017.03.017)
Supplement: Table S1 [file mmc2.pdf]

Supplementary Table S1. Parameters relating to twin modelling for ERG responses. The Akaike information criteriertion (AIC) is used to evaluate the most parsimonious, best fitting model (identified by the lowest or most negative AIC), which is highlighted in bold. For each model the "minus 2 log-likelihood" (minus2ll), and degrees of freedom (df) are given. A = additive genetic effects, D = dominant genetic effects, C = common environmental effects and E = unique environmental effects (and measurement error).

Stimulus	Response component	Parameter	Model	Comparison	minus2LL	df	AIC
Scotopic dim flash	b-wave	Amplitude	ACE	-	2100.25	202	1686.25
			ADE	ACE	2099.74	202	1695.74
			**AE**	**ACE**	**2100.25**	**203**	**1694.25**
			E	ACE	2134.89	204	1726.89
		Implicit time	ACE	-	1588.20	202	1184.20
			ADE	ACE	1588.13	202	1184.13
			AE	ACE	1588.20	203	1182.20
			**E**	**ACE**	**1589.19**	**204**	**1181.19**
Scotopic standard flash	a-wave	Amplitude	ACE	-	1965.51	202	1561.51
			ADE	ACE	1966.56	202	1562.56
			**AE**	**ACE**	**1966.56**	**203**	**1560.56**
			E	ACE	2013.45	204	1605.45
		Implicit time	ACE	-	875.85	202	471.85
			**ADE**	**ACE**	**873.28**	**202**	**469.28**
			AE	ACE	875.85	203	469.85
			E	ACE	891.57	204	483.57
	b-wave	Amplitude	ACE	-	2184.55	202	1780.55
			ADE	ACE	2184.72	202	1780.72
			**AE**	**ACE**	**2184.72**	**203**	**1778.72**
			E	ACE	2248.47	204	1840.47
		Implicit time	ACE	-	1037.56	202	633.56
			ADE	ACE	1036.03	202	632.03
			**AE**	**ACE**	**1037.56**	**203**	**631.56**
			E	ACE	1053.78	204	645.78
	b-to-a ratio		ACE	-	32.80	202	-371.20
			ADE	ACE	33.42	202	-370.58
			**AE**	**ACE**	**33.42**	**203**	**-372.58**
			E	ACE	78.40	204	-329.60
Scotopic bright flash	a-wave	Amplitude	ACE	-	1995.54	201	1593.54
			ADE	ACE	1995.73	201	1593.73
			**AE**	**ACE**	**1995.73**	**202**	**1591.73**
			E	ACE	2042.58	203	1636.58
		Implicit time	ACE	-	532.05	201	130.05
			ADE	ACE	530.28	201	128.28
			**AE**	**ACE**	**532.05**	**202**	**128.05**
			E	ACE	553.51	203	147.51
	b-wave	Amplitude	ACE	-	2174.48	201	1772.48
			ADE	ACE	2174.74	201	1772.74
			**AE**	**ACE**	**2174.74**	**202**	**1770.74**
			E	ACE	2233.85	203	1827.85
		Implicit time	ACE	-	1123.96	201	721.96
			**ADE**	**ACE**	**1121.82**	**201**	**719.82**
			AE	ACE	1123.96	202	719.96
			E	ACE	1142.79	203	736.79
	b-to-a ratio		ACE	-	-92.45	201	-494.45
			ADE	ACE	-92.51	201	-494.51
			**AE**	**ACE**	**-92.45**	**202**	**-496.45**
			E	ACE	-28.06	203	-434.06
Photopic 30 Hz flicker	Peak	Amplitude	ACE	-	1643.04	188	1267.04
			ADE	ACE	1644.48	188	1268.48
			**AE**	**ACE**	**1644.48**	**189**	**1266.48**

			E	ACE	1680.28	190	1300.28
		Implicit time	ACE	-	612.57	189	234.57
			ADE	ACE	612.55	189	234.55
			**AE**	**ACE**	**612,57**	**190**	**232.57**
			E	ACE	634.99	191	252.99
Photopic single flash	a-wave	Amplitude	ACE	-	1034.80	174	686.80
			ADE	ACE	1035.59	174	687.59
			**AE**	**ACE**	**1035.59**	**175**	**685.59**
			E	ACE	1107.04	176	755.04
		Implicit time	ACE	-	325.37	174	-22.63
			ADE	ACE	325.37	174	-22.63
			**AE**	**ACE**	**325.37**	**175**	**-24.63**
			E	ACE	331.88	176	-20.12
	b-wave	Amplitude	ACE	-	1571.62	174	1223.62
			ADE	ACE	1571.63	174	1223.63
			**AE**	**ACE**	**1571.63**	**175**	**1221.63**
			E	ACE	1640.33	176	1288.33
		Implicit time	ACE	-	496.59	174	148.59
			ADE	ACE	496.62	174	148.62
			**AE**	**ACE**	**496.62**	**175**	**146.62**
			E	ACE	525.21	176	173.21
	b-to-a ratio		ACE	-	311.99	174	-36.01
			ADE	ACE	311.98	174	-36.02
			**AE**	**ACE**	**311.99**	**175**	**-38.01**
			E	ACE	367.13	176	15.13
PhNR	Amplitude at 65 ms		ACE	-	3063.28	154	2755.28
			ADE	ACE	2086.56	154	2778.56
			**AE**	**ACE**	**3063.28**	**155**	**2753.28**
			E	ACE	3079.32	156	2767.32
	Amplitude at trough		ACE	-	3093.13	154	2785.13
			ADE	ACE	3123.29	154	2815.29
			**AE**	**ACE**	**3093.13**	**155**	**2783.13**
			E	ACE	3118.71	156	2806.71
	Implicit time of trough		ACE	-	1026.11	154	718.11
			ADE	ACE	1024.54	154	716.54
			**AE**	**ACE**	**1026.11**	**155**	**716.11**
			E	ACE	1057.23	156	745.23
	PhNR/b-wave		ACE	-	517.95	128	261.95
			ADE	ACE	-394.03	128	-650.03
			**AE**	**ACE**	**-394.03**	**129**	**-652.03**
			E	ACE	-361.12	130	-621.12
i-wave	Amplitude		ACE	-	3110.77	154	2802.77
			ADE	ACE	3137.98	154	2829.98
			**AE**	**ACE**	**3111.28**	**155**	**2801.28**
			E	ACE	3169.86	156	2857.86
	Implicit time		ACE	-	621.66	154	313.66
			ADE	ACE	622.05	154	314.05
			**AE**	**ACE**	**622.05**	**155**	**312.05**
			E	ACE	641.89	156	319.89
